# Does Size Outweigh Number in Predicting Survival After Pulmonary Metastasectomy for Soft Tissue Sarcoma? Insights from a Retrospective Multicenter Study

**DOI:** 10.1245/s10434-025-17450-2

**Published:** 2025-05-14

**Authors:** Anton Burkhard-Meier, Matthias Grube, Vindi Jurinovic, Abbas Agaimy, Markus Albertsmeier, Luc M. Berclaz, Dorit Di Gioia, Hans Roland Dürr, Rüdiger von Eisenhart-Rothe, Chukwuka Eze, Katja Fechner, Emma Fey, Sinan E. Güler, Judith S. Hecker, Anne Hendricks, Felix Keil, Alexander Klein, Carolin Knebel, Julia R. Kovács, Wolfgang G. Kunz, Ulrich Lenze, Alisa M. Lörsch, Mathias Lutz, Norbert Meidenbauer, Carolin Mogler, Sebastian Schmid, Nina-Sophie Schmidt-Hegemann, Christian Schneider, Sabine Semrau, Wulf Sienel, Martin Trepel, Johannes Waldschmidt, Armin Wiegering, Lars H. Lindner

**Affiliations:** 1 Department of Medicine III, University Hospital, LMU Munich, Munich, Germany; 2 Bavarian Cancer Research Center (BZKF), Erlangen, Germany; 3 Department of Internal Medicine III, University Hospital Regensburg, Regensburg, Germany; 4 Institute for Medical Information Processing, Biometry, and Epidemiology, University Hospital, LMU Munich, Munich, Germany; 5 Institute of Pathology, University of Erlangen-Nuremberg, Erlangen, Germany; 6 Department of General, Visceral and Transplantation Surgery, University Hospital, LMU Munich, Munich, Germany; 7 Department of Orthopedics and Trauma Surgery, University Hospital, LMU Munich, Munich, Germany; 8 Department of Orthopaedics and Sports Orthopaedics, Klinikum rechts der Isar, Technical University of Munich, Munich, Germany; 9 Department of Radiation Oncology, University Hospital, LMU Munich, Munich, Germany; 10 Department of Surgery, University of Erlangen-Nuremberg, Erlangen, Germany; 11 Department of Medicine III, School of Medicine and Health, Technical University of Munich, Munich, Germany; 12 TranslaTUM, Center for Translational Cancer Research, Technical University of Munich (TUM), Munich, Germany; 13 Department of General, Visceral, Transplantation, Vascular and Pediatric Surgery, University Hospital Würzburg, Würzburg, Germany; 14 Institute of Pathology, University Regensburg, Regensburg, Germany; 15 Department of Thoracic Surgery, University Hospital, LMU Munich, Munich, Germany; 16 Department of Radiology, University Hospital, LMU Munich, Munich, Germany; 17 Department of Medicine II, Hematology and Oncology, University Hospital of Augsburg, Augsburg, Germany; 18 Department of Medicine 5, University of Erlangen-Nuremberg, Erlangen, Germany; 19 Institute of Pathology, School of Medicine and Health, Technical University Munich, Munich, Germany; 20 Department of Trauma Surgery, University Hospital of Augsburg, Augsburg, Germany; 21 Department of Radiation Oncology, University of Erlangen-Nuremberg, Erlangen, Germany; 22 Department Internal Medicine II, University Hospital Würzburg, Würzburg, Germany; 23 Department of General, Visceral, Transplant, and Thoracic Surgery, University Hospital of Frankfurt, Frankfurt, Germany

**Keywords:** Soft tissue sarcoma, Metastasis, Pulmonary metastasectomy, Systemic therapy, Leiomyosarcoma, Synovial sarcoma, Undifferentiated pleomorphic sarcoma

## Abstract

**Background:**

Pulmonary metastasectomy (PM) is the most frequently performed local ablative therapy for leiomyosarcoma (LMS), synovial sarcoma (SyS), and undifferentiated pleomorphic sarcoma (UPS). This study aimed to assess surgical feasibility, outcome, and clinical prognostic factors, as well as the value of a peri-interventional systemic therapy.

**Methods:**

This multicenter retrospective study enrolled 77 patients with LMS, SyS, or UPS who underwent first-time complete resection of isolated lung metastases between 2009 and 2021. Disease-free survival (DFS), overall survival (OS), and clinical prognostic factors were analyzed.

**Results:**

After the first PM, the median DFS was 7.4 months, and the median OS was 58.7 months. A maximal lesion diameter greater than 2 cm was associated with reduced DFS in both the univariable (hazard ratio [HR], 2.29; *p* = 0.006) and multivariable (HR, 2.60; *p* = 0.005) analyses. The univariable analysis identified a maximal lesion diameter greater than 2 cm as an adverse prognostic factor for OS (HR, 5.6; *p* < 0.001), whereas a treatment-free interval longer than 12 months was associated with improved OS (HR, 0.42; *p* = 0.032). The addition of systemic therapy was associated with a trend toward improved DFS for patients with lesions larger than 2 cm (HR, 0.29; *p* = 0.063). Severe postoperative complications (grade ≥IIIa) occurred in 2 % of the patients.

**Conclusion:**

The size of resected lung metastases might be a more relevant prognostic factor than their number for patients with LMS, SyS, or UPS. For patients with lung metastases larger than 2 cm in maximal diameter, additional systemic therapy may be warranted.

**Supplementary Information:**

The online version contains supplementary material available at 10.1245/s10434-025-17450-2.

Soft tissue sarcomas (STSs) constitute a heterogeneous group of mesenchymal malignancies accounting for approximately 1 % of all cancers in adults.^1^ Up to half of STS patients experience metastatic disease, with the lungs as the most commonly affected site.^2,3^ Leiomyosarcoma (LMS), synovial sarcoma (SyS), and undifferentiated pleomorphic sarcoma (UPS) are among the most common histologic subtypes with lung metastasis.^4^

The prognosis for patients with metastatic STS remains poor, as standard anthracycline-based chemotherapy provides survival rates of only 1–2 years.^5,6^ However, retrospective analyses indicate that pulmonary metastasectomy (PM) can provide survival benefits and even long-term survival for selected patients.

In 1997, a retrospective analysis of 5206 patients from the International Registry of Lung Metastases (IRLM) who underwent PM reported a 5 year survival of 32 % among STS and bone sarcoma patients (*n* = 2173).^7^ Two more recent systematic reviews encompassing 13 retrospective studies with a total of 1282 STS patients reported 5-year survival rates ranging from 18 to 58 %.^4,8^ Previous studies have identified a small number or size of lesions, the absence of extrapulmonary metastasis, a long disease-free interval, and a complete resection as potential prognostic factors.^4,9–11^

The current National Comprehensive Cancer Network (NCCN) guidelines do not specify strict rules for metastasectomy with or without systemic therapy. Instead, treatment decisions should consider several factors, including resectability, the number and location of metastases, and the patient’s performance status.^12^ The German guidelines for STS recommend PM after a multidisciplinary tumor board discussion of patients with resectable metastases, provided the primary tumor is controlled. Previous PMs are not considered a contraindication, and systemic therapy is not recommended after metachronous PM.^13^ However, it remains unclear whether certain patient subgroups derive benefit from additional systemic therapy.

Concerning histologic subtypes, no significant differences in the application of PM have been identified. A previous study demonstrated improved outcomes after PM for LMS compared with other subtypes, primarily SyS and UPS.^14^

Previously, our study group of the Bavarian Cancer Research Center (BZKF) performed a large-scale retrospective study of patients with metastatic STS and local ablative therapy, including surgical metastasectomies, after tumor board recommendation. This longitudinal study, characterized by high heterogeneity in histologic subtypes and types of local ablative therapies, reported a median overall survival (OS) from first metastasis of 5.4 years in a cohort of 246 patients. A treatment-free interval (TFI) of 12 months or longer and treatment of hepatic and other extrapulmonary metastasis were associated with improved survival, whereas rare histologic subtypes and local therapies other than surgery and stereotactic radiotherapy represented poor prognostic factors.^3^

The current multicenter study examined feasibility, outcomes, and potential clinical prognostic factors for patients with LMS, SyS, or UPS who underwent first-time complete resection of isolated lung metastases.

## Materials and Methods

### Patient Selection and Data Extraction

An exploratory retrospective multicenter study was performed at six university hospitals in Germany: Ludwig Maximilian University (LMU) of Munich, Technical University (TU) of Munich, Augsburg, Erlangen, Regensburg, and Würzburg. The study enrolled patients 18 years of age or older who had pathologically confirmed LMS, SyS, or UPS and had received first-time PM for isolated pulmonary metastases between June 2009 and December 2021. Patients who had metastasectomy without achieving complete resection (R0) of all known lesions were excluded. No specific limits on the number or size of metastatic lesions were defined.

The treatment strategy for all the patients was discussed in the local multidisciplinary sarcoma tumor board. The surgical approach (video-assisted thoracoscopic surgery [VATS] or anterolateral thoracotomy) was determined based on the location, size, and quantity of the metastases. Generally, VATS was performed for peripheral and limited lesions, whereas anterolateral thoracotomy was preferred for multiple or centrally located metastases to allow manual palpation and ensure complete resection.

Lymph node sampling was performed at the discretion of the surgeon. For thoracotomy, an epidural catheter was routinely placed for postoperative pain management. Follow-up evaluations were performed according to international guideline recommendations.^15^

Clinical, pathologic, and outcomes data were extracted from the prospectively maintained databases of the respective institutions. At first diagnosis, the current World Health Organization (WHO) tumor classification system and the French grading system were applied.^16,17^ Dates of death were determined with the help of the Cancer Registry of Bavaria. At all study sites, data were collected using the biomedical research portal CentraXX (KAIROS GmbH, Bochum, Germany) in accordance with local security standards.

### Outcomes

The primary objective of this study was to investigate the impact of PM and prognostic clinical factors on patients with metastatic LMS, UPS, or SyS. The endpoints of this analysis were disease-free survival (DFS) and overall survival (OS). DFS was calculated as the time from the first PM to either relapse in any organ site or death of any cause. OS was estimated as the time from the first PM to death from any cause. Two-stage PM was considered as one surgical procedure for statistical analysis. Primary tumor control was defined as absence of progression or new primary tumor/local recurrence at the date of PM. Treatment-free interval (TFI) was defined as the time between the end of any last therapy at previous tumor diagnosis/progression/recurrence and the start of PM. Synchronous metastasis was defined as the presence of metastatic disease identified during the initial diagnostic workup at the time of the primary tumor diagnosis.

Postoperative complications up to 30 days after PM were classified using the Clavien-Dindo classification.^18^ Response evaluation criteria in solid tumors (RECIST) v1.1 were used to evaluate the efficacy of additional systemic therapy.^19^ The number and size of metastases were obtained from the pathologic reports. In patients with preoperative treatment, measurements reflect the status after treatment at the time of surgery.

### Statistical Analysis

Both OS and DFS were analyzed with Cox proportional hazards regression. The results with a *p* value of 0.05 or lower were considered statistically significant. To determine whether specific subgroups of patients benefit from additional systemic therapy, we performed interaction analyses between systemic therapy and various clinical factors. Statistical analysis was performed using R software version 4.0.3 (R Foundation for Statistical Computing, Vienna, Austria).

### Ethics

The internal review board and the ethics committee at the LMU University Hospital of Munich, Germany approved the study protocol (protocol no. 22-0822). In addition, the respective local ethics committees at each study site approved the current study.

## Results

### Patient Cohort and Treatment

The study cohort included 77 patients. The patient demographics and disease characteristics are presented in Table 1. The median age was 54 years, and 61 % (*n* = 47) of the patients were female. The histologic subtypes were evenly distributed as follows: LMS (*n* = 27, 35 %), SyS (*n* = 25, 32 %), and UPS (*n* = 25, 32 %).

**Table 1:** Baseline characteristics

Factor	Strata	*N*	%
Total		77	100
Age at initial diagnosis (years)	Median (range)	54 (19–81)	
Sex	Male	30	39
	Female	47	61
Grading according to FNCLCC	G2	31	40
	G3	46	60
Histologic subtype	LMS	27	35
	SyS	25	32
	UPS	25	32
Site of primary tumor	Extremity	45	58
	Non-extremity	32	42
Primary tumor size (cm)	≤10	50	65
	>10	27	35
Radiotherapy at first diagnosis	Yes	41	53
	No	36	47
Systemic therapy at first diagnosis	Yes	49	64
	No	28	36
First occurrence of pulmonary metastasis	Synchronous	13	17
	Metachronous	64	83
Treatment-free interval before first PM (months)	<12	41	53
	≥12	36	47
Type of PM	VATS	24	31
	Anterolateral thoracotomy	53	69
No. of treated metastases	1	37	48
	2–5	27	35
	>5	13	17
Bipulmonary metastases	Yes	21	27
	No	56	73
Largest diameter of treated metastases (cm)	≤2	60	78
	>2	17	22
Primary tumor control at date of PM	Yes	71	92
	No	6	8
Additional systemic therapy	Yes	23	30
	No	54	70

*FNCLCC* Fédération Nationale des Centres de Lutte Contre le Cancer, *LMS* leiomyosarcoma, *SyS* synovial sarcoma, *UPS* undifferentiated pleomorphic sarcoma, *PM* pulmonary metastasectomy, *VATS* video-assisted thoracoscopic surgery

Pulmonary metastasectomies were performed for a median number of two pulmonary metastases (range, 1–16), with the largest metastasis having a median diameter of 1.2 cm (range, 0.3–11.2 cm). In 31 % of PMs (*n* = 24), VATS was performed, whereas 69 % (*n* = 53) of the patients underwent anterolateral thoracotomy. Additional lymph node dissection or sampling was performed for 57 % of the patients (*n* = 44), with no lymph node involvement detected in any case. The median hospital length of stay was 8 days (range, 4–22 days).

### Postoperative Complications of PM

For 84 % of the patients (*n* = 65), no postoperative complications were reported. According to the Clavien-Dindo classification, 4 % (*n* = 3) of the patients experienced grade I complications, 10 % (*n* = 8) had grade II complications, and 1 % (*n* = 1) had a grade IIIa complication. Additionally, three patients experienced a second postoperative complication (grade II: 3 %, *n* = 2; grade IIIb: 1 %, *n* = 1). Grade I complications included apical pneumothoraxes (*n* = 2) and a seroma (*n* = 1). Grade II complications consisted of infections (*n* = 5), bleedings (*n* = 2), pain exacerbation (*n* = 1), and complications not further specified (*n* = 2). The grade IIIa complication was a pneumothorax (*n* = 1), and the grade IIIb complication was a pleural empyema (*n* = 1). All the reported postoperative complications occurred after thoracotomy.

### DFS and OS After PM

During a median follow-up period of 43.2 months (95 % confidence interval [CI], 35.7–60.0), the median DFS after the first PM was 7.4 months (95 % CI, 6.5–13.4 months), and the median OS was 58.7 months (95 % CI, 46.7–NA). The 2 year OS rate was 85.8 % (95 % CI, 78.0–94.4 %), and the 5 year OS rate was 49.3 % (95 % CI, 35.9–67.7 %). By the end of follow-up period, 63 DFS events and 29 deaths were reported. For the patients with synchronous metastasis, the median DFS after the first PM was 6.4 months (95 % CI, 4.6–NA), and the median OS was 46.7 months (95 % CI, 29.3–NA).

### Prognostic Factors for Patients After PM

**Fig. 1:**
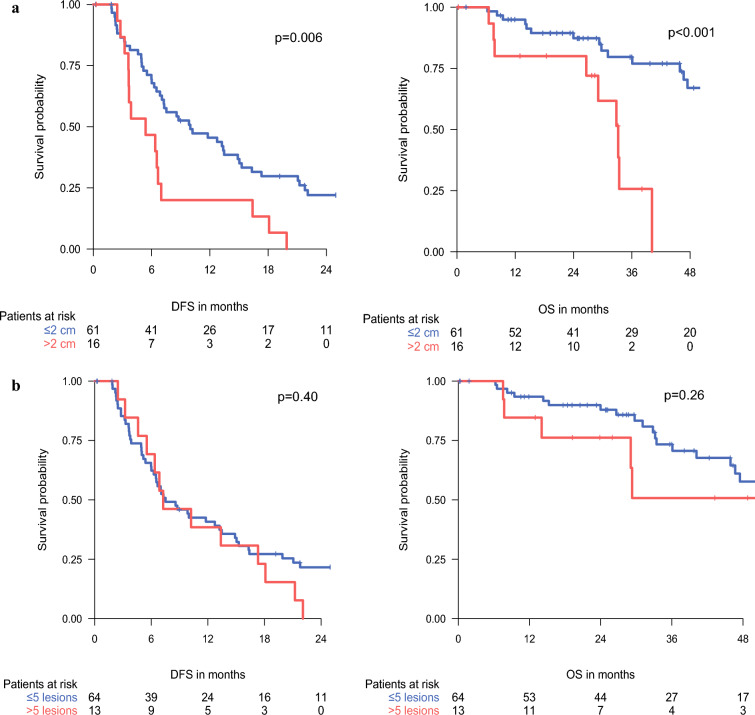
Disease-free survival (DFS) and overall survival (OS) after pulmonary metastasectomy (PM) according to **a** largest diameter and **b** number of treated lesions

The analyses of clinical variables and their impact on DFS and OS are summarized in Tables 2 and 3. Uni- and multivariable analyses identified a maximal lesion diameter larger than 2 cm as a poor prognostic factor for DFS after PM, whereas all other tested clinical variables were not significantly associated with DFS (Fig. 1).

**Table 2:** Prognostic factors for disease-free survival (DFS) after pulmonary metastasectomy (PM) according to uni- and multivariable analysis

Factor	Strata	Univariable		Multivariable	
		*p* Value	HR (95 % CI)	*p* Value	HR (95 % CI)
Age (years)	≤60 versus >60	0.51	0.83 (0.48–1.45)	0.59	0.83 (0.42–1.64)
Sex	Female versus male	0.56	0.86 (0.51–1.43)	0.33	0.74 (0.40–1.37)
Histologic subtype	SyS versus LMS	0.39	0.77 (0.42–1.40)	0.37	0.75 (0.40–1.40)
	UPS versus LMS	0.76	0.91 (0.49–1.67)	0.49	0.75 (0.33–1.71)
Grading	G3 versus G2	0.54	0.86 (0.52–1.41)	0.14	0.62 (0.33–1.18)
Treatment-free interval (months)	≥12 versus <12	0.12	0.68 (0.41–1.11)	0.17	0.63 (0.32–1.23)
Timing of metastasis	Metachronous versus synchronous	0.67	0.87 (0.45–1.67)	0.99	1.0 (0.31–3.16)
Primary tumor control	Yes versus no	0.50	0.73 (0.29–1.83)	0.74	0.84 (0.29–2.40)
Systemic therapy	Yes versus no	0.68	1.12 (0.65–1.93)	0.27	1.69 (0.67–4.29)
No. of treated lesions	>5 versus ≤5	0.40	1.30 (0.70–2.41)	0.42	1.35 (0.65–2.81)
Maximal diameter of treated lesions (cm)	>2 versus ≤2	**0.006**	2.29 (1.27–4.14)	**0.005**	2.60 (1.34–5.03)

*HR* hazard ratio, *CI* confidence interval, *SyS* synovial sarcoma, *LMS* leiomyosarcoma, *UPS* undifferentiated pleomorphic sarcoma

Bold values indicate statistical significance at the *p* < 0.05 level

**Table 3:** Prognostic factors for overall survival (OS) after pulmonary metastasectomy (PM) according to univariable analysis

Factor	Strata	*p* Value	HR (95 % CI)
Age (years)	≤60 versus >60	0.53	0.77 (0.33–1.76)
Sex	Female versus male	*0.063*	0.48 (0.22–1.04)
Histologic subtype	SyS versus LMS	0.71	1.19 (0.47–3.03)
	UPS versus LMS	*0.056*	2.45 (0.98–6.16)
Grading	G3 versus G2	0.98	1.01 (0.47–2.17)
Treatment-free interval (months)	≥12 versus <12	**0.032**	0.42 (0.19–0.93)
Timing of metastasis	Metachronous versus synchronous	0.37	0.63 (0.23–1.72)
Primary tumor control	Yes versus no	0.65	0.72 (0.17–3.08)
Systemic therapy	Yes versus no	0.12	1.84 (0.86–3.94)
No. of treated lesions	>5 versus ≤5	0.26	1.69 (0.68–4.19)
Maximal diameter of treated lesions (cm)	>2 versus ≤2	**<0.001**	5.60 (2.28–13.74)

*HR* hazard ratio, *CI* confidence interval, *SyS* synovial sarcoma, *LMS* leiomyosarcoma, *UPS* undifferentiated pleomorphic sarcoma

Bold values indicate statistical significance at the *p* < 0.05 level

Italic values indicate *p* < 0.1

In a multivariable analysis using continuous variables for size and number of treated lesions, the largest size showed a trend toward reduced DFS (hazard ratio [HR], 1.13; *p* = 0.054), whereas the number of treated lesions did not significantly influence DFS (HR,1.02; *p* = 0.58; Supplementary File 1). Regarding OS, a TFI of 12 months or longer was identified as a favorable prognostic factor, whereas a maximal lesion diameter larger than 2 cm was negatively associated in the univariable analysis. Furthermore, UPS histology and male sex were non-significantly associated with reduced OS.

### PM and Systemic Therapy

In the study cohort, 30 % (*n* = 23) received systemic therapy in addition to PM at the respective tumor diagnosis or recurrence. The most common regimen was anthracycline + ifosfamide (AI) ± regional hyperthermia (RHT). Systemic therapy was applied before PM for 52 % (*n* = 12), before and after PM for 35 % (*n* = 8), and after PM for 13 % (*n* = 3) of the patients. A response to systemic induction therapy according to RECIST or other response criteria according to tumor board report was observed in 71 % (*n* = 15) of evaluable patients.

An interaction analysis of the use of systemic therapy and the other assessed clinical factors regarding an impact on DFS and OS was performed (Table 4). The maximal diameter of treated lesions larger than 2 cm had a significant interaction with an additional systemic therapy (*p* = 0.040). An additional subgroup analysis showed that systemic therapy tended to improve DFS for the patients with a maximal diameter of treated lesions larger than 2 cm (HR, 0.29; 95 % CI, 0.078–1.07; *p* = 0.063).

**Table 4:** Interaction analysis between various clinical variables and additive systemic therapy with regard to disease-free survival (DFS) and overall survival (OS) (*p* values of interaction analysis)

Factor	Strata	DFS	OS
		*p* Value	*p* Value
Age (years)	≤60 versus >60	0.11	0.62
Sex	Female versus male	0.47	0.65
Grading	G3 versus G2	0.57	0.41
Treatment-free interval (months)	≥12 versus <12	0.86	0.97
Timing of metastasis	Metachronous versus synchronous	NA	NA
Primary tumor control	Yes versus no	0.77	0.15
No. of treated lesions	>5 versus ≤5	0.45	0.36
Maximal diameter of treated lesions (cm)	>2 versus ≤2	**0.040**	*0.098*

*DFS* disease-free survival, *OS* overall survival, *NA* not available

Bold values indicate statistical significance at the *p* < 0.05 level

Italic values indicate *p* < 0.1

## Discussion

This study was a multicenter analysis of 77 patients with metastatic LMS, UPS, or SyS who underwent PM. By focusing on the three most common histologic subtypes among STS lung metastases and including only patients who underwent first-time complete surgical resection of isolated lung metastases, this subgroup analysis offers valuable clinical insights into a relatively homogeneous cohort. Additionally, the multicenter design reduced the risk of center-specific biases.^20^

The lungs represent the most common site of metastasis in STS, and PM is the most frequently performed metastasis-directed local ablative therapy. Consistent with previous data, complications after PM in this cohort were predominantly non-severe.^21,22^ Notably, no postoperative complications were observed in patients treated with VATS. However, the low incidence of non-severe complications in our study might be attributed to the common under-reporting in this category.^23^ Given the comparable survival rates after stereotactic body radiotherapy (SBRT),^3,24^ factors such as patients’ quality of life and economic considerations should be incorporated into treatment decisions. In our study, the median hospital length of stay was 8 days, in contrast to SBRT, which typically is performed in an outpatient setting. Future prospective studies should evaluate these aspects to guide personalized treatment decisions.

In our study, the median DFS after first-time PM was 7.4 months, and the median OS was 58.7 months, with a 5 year survival rate of 49.3 %. This result falls within the higher range of previously reported 5 year survival rates ranging from 18 to 58 %.^4,8^

The long OS observed in our cohort could be attributed to the sole inclusion of chemosensitive histologic subtypes,^13^ but also may reflect the impact of treatment in specialized institutions.^25,26^ The relatively short median DFS compared with the OS further highlights the value of repeat PMs, as previously demonstrated in other studies.^14,27^

The maximal diameter of treated lesions larger 2 cm emerged as the strongest adverse prognostic factor for DFS and OS after first-time PM. In contrast, no significant differences in survival were observed regarding the number of treated lesions, histologic subtype, or time point of metastasis. Analyzing the number and size of treated lesions as continuous variables confirmed the substantial impact of size on DFS, whereas the number of treated lesions showed no significant effect.

Previous studies, which often included multiple STS subtypes, have variably identified lesion size and number of treated lesions as prognostic factors.^4^ In one study focusing on LMS, a metastasis size larger than 2 cm negatively influenced PFS, whereas the number of treated lesions did not significantly affect survival after PM.^11^ Furthermore, the diameter of the largest pulmonary metastasis has been proposed as a negative prognostic factor for STS patients who have isolated pulmonary metastases treated with first-line systemic therapy.^28^

Although oligometastatic disease typically is defined by a limited number of metastases (e.g., up to 5 metastases in 3 organ sites),^29^ our findings suggest that lesion size might be a more critical factor in the assessment of PM. Historically, tumor board decisions may have prioritized metastasis count over size when defining the stage of metastasis.

In the multivariable analysis, histologic subtype did not significantly influence DFS. However, the univariable analysis indicated a tendency toward poorer OS for UPS. This might have been related to the higher chemosensitivity and more systemic options for patients with SyS and LMS.^30,31^ The small size of the subgroups precluded definitive conclusions on histotype-specific differences and requires further studies.

In our study, TFI did not significantly impact DFS, which could have been due to limited statistical power, but also might reflect the encouraging outcomes observed for patients with synchronous metastasis and a short TFI. In our large-scale study of local ablative therapies in metastatic STS, a TFI of 12 months or longer emerged as the most consistent prognostic factor.^3^ Specifically for PM, the high prognostic value of a long disease-free interval has been confirmed in the systematic review by Stamenovic et al.^4^

One of the main remaining questions with respect to local ablative therapies in STS is the role of additional systemic therapy. Previous studies provided varying results, with either no impact or even a negative impact by the combination with systemic therapy.^32–35^ In our previous study, we could demonstrate a PFS benefit for patients younger than 60 years with four or more treated metastases from the combination of systemic therapy with various local ablative therapies. This effect was more pronounced when the maximal diameter was larger than 2 cm.^3^ In the current analysis of patients with first-time PM, additional systemic therapy non-significantly (*p* = 0.063) improved DFS for the patients with a maximal diameter of treated lesions larger than 2 cm.

This analysis was limited by the small number of patients with additional systemic therapy and should be repeated with a larger sample. Moreover, 57 % of the patients receiving systemic therapy presented with synchronous metastasis, reflecting a different clinical scenario compared with oligorecurrence.

Further limitations of this study included its retrospective design and the absence of a control cohort. Due to the limited sample size, a multivariate analysis for OS was not possible. Our findings may not be generalizable to the broader STS population because our study focused on a highly selected cohort of patients who underwent thorough evaluation by specialized tumor boards before PM recommendation.

To validate our findings, we have initiated a prospective registry study enrolling all patients with newly diagnosed metastatic STS regardless of the treatment (DRKS00035722). Prospective registries ensure more accurate data collection, minimize selection bias, and are particularly suited for studying rare diseases such as STS.

## Conclusion

Our multicenter analysis provides significant information about PM for patients with three of the most common histologic subtypes in metastatic STS. The postoperative complication rate was low, and long-term survival after PM was achieved for up to half of the patients. A maximal lesion diameter larger than 2 cm emerged as the strongest adverse prognostic factor, whereas the number of metastases had less impact on outcomes in our cohort. Patients with a maximal lesion diameter larger than 2 cm may benefit from additional systemic therapy.

## Supplementary Information

Below is the link to the electronic supplementary material.

Supplementary file1 (DOCX 20 KB)
