# Supplementary material for: Does Size Outweigh Number in Predicting Survival After Pulmonary Metastasectomy for Soft Tissue Sarcoma? Insights from a Retrospective Multicenter Study
Source: Ann Surg Oncol. 2025 May 14;32(8):5948–56. doi: 10.1245/s10434-025-17450-2 (PMC12222427; doi:10.1245/s10434-025-17450-2)
Supplement: Supplementary file 1 — Supplementary file1 (DOCX 20 KB) [file 10434_2025_17450_MOESM1_ESM.docx]

**Supplementary File**

File 1: Prognostic factors for disease-free survival (DFS) after pulmonary metastasectomy (PM), uni- and multivariable analysis with number and maximal diameter of treated lesions as continuous variables

|  |  | **Univariable** | | **Multivariable** | |
| --- | --- | --- | --- | --- | --- |
| **Factor** | **Strata** | **p-value** | **HR (95% CI)** | **p-value** | **HR (95% CI)** |
| Age | ≤60 vs. >60 | 0.51 | 0.83 (0.48-1.45) | 0.74 | 0.89 (0.45-1.77) |
| Sex | Female vs. male | 0.56 | 0.86 (0.51-1.43) | 0.50 | 0.81 (0.44-1.49) |
| Histological subtype | SyS vs. LMS | 0.39 | 0.77 (0.42-1.40) | 0.37 | 0.75 (0.40-1.41) |
|  | UPS vs. LMS | 0.76 | 0.91 (0.49-1.67) | 0.41 | 0.71 (0.31-1.62) |
| Grading | G3 vs. G2 | 0.54 | 0.86 (0.52-1.41) | 0.30 | 0.72 (0.39-1.33) |
| Treatment-free interval (months) | ≥12 vs. <12 | 0.12 | 0.68 (0.41-1.11) | 0.12 | 0.59 (0.30-1.15) |
| Timing of metastasis | Metachronous vs. synchronous | 0.67 | 0.87 (0.45-1.67) | 0.55 | 1.42 (0.46-4.40) |
| Primary tumor control | Yes vs. No | 0.50 | 0.73 (0.29-1.83) | 0.55 | 0.72 (0.24-2.12) |
| Systemic therapy | Yes vs. No | 0.68 | 1.12 (0.65-1.93) | 0.58 | 0.78 (0.32-1.88) |
| Number of treated lesions | Continuous | 0.50 | 1.03 (0.96-1.09) | 0.58 | 1.02 (0.94-1.11) |
| Maximal diameter of treated lesions (cm) | Continuous | *0.057* | 1.09 (1.0-1.20) | *0.054* | 1.13 (1.0-1.29) |

LPS: Liposarcoma, LMS: Leiomyosarcoma, SyS: Synovial sarcoma, UPS: Undifferentiated pleomorphic sarcoma

File 2: Interaction analysis between various clinical variables and additive systemic therapy (p values of interaction analysis)

|  |  | **DFS** | **OS** |
| --- | --- | --- | --- |
| **Factor** | **Strata** | **p-value** | **p-value** |
| Age | ≤60 vs. >60 | 0.11 | 0.62 |
| Sex | Female vs. male | 0.47 | 0.65 |
| Grading | G3 vs. G2 | 0.57 | 0.41 |
| Treatment-free interval (months) | ≥12 vs. <12 | 0.86 | 0.97 |
| Timing of metastasis | Metachronous vs. synchronous | NA | NA |
| Primary tumor control | Yes vs. No | 0.77 | 0.15 |
| Number of treated lesions | >5 vs. ≤5 | 0.45 | 0.36 |
| Maximal diameter of treated lesions (cm) | >2 vs. ≤2 | **0.040** | *0.098* |

DFS: Disease-free survival, OS: Overall survival, NA: Not available
